# The effects of intraday operation time on pain and anxiety of patients undergoing septoplasty

**DOI:** 10.1016/j.bjorl.2019.09.006

**Published:** 2019-10-31

**Authors:** Serkan Kayabasi, Serkan Cayir, Omer Hizli

**Affiliations:** a Aksaray University, Faculty of Medicine, Department of ENT, Aksaray, Turkey; b Aksaray University, Aksaray Education and Research Hospital, Department of ENT, Aksaray, Turkey; c Giresun University, Prof Dr. A. Ilhan Ozdemir Education and Research Hospital, Department of ENT, Giresun, Turkey

**Keywords:** Septoplasty, Anxiety, Postoperative pain, Operation time

## Abstract

**Introduction:**

Anxiety and pain levels of septoplasty patients may vary according to intraday operation time.

**Objective:**

To investigate the effects of septoplasty operation and intraday operation time on anxiety and postoperative pain.

**Methods:**

Ninety-eight voluntary patients filled out the hospital anxiety and depression scale to measure the anxiety level three weeks before, one hour before and one week after surgery. Forty-nine patients were operated at 8:00 am (morning group); other 49 were operated at 03:00 pm (afternoon group). We used a visual analogue scale to measure postoperative pain. Preoperative and postoperative scores were compared, as were the scores of the groups.

**Results:**

Median hospital anxiety and depression scale scores one hour before the operation [6 (2–10)] were significantly higher compared to the median scores three weeks before the operation [3 (1–6)] (*p* <  0.001), and one week after the operation [2 (1–6)] were significantly lower compared to the median scores three weeks before the operation [3 (1–6)] (*p* <  0.001). Hospital anxiety and depression scale scores one hour before the operation were significantly greater in the afternoon group [8 (7–10)], compared to the morning group [4 (2–6)] (*p* <  0.001). Postoperative first, sixth, twelfth and twenty-fourth-hour pain visual analogue scale scores were significantly higher in the afternoon group compared to the morning group (*p* <  0.001).

**Conclusion:**

Septoplasty might have an increasing effect on short-term anxiety and postoperative pain. Performing this operation at a late hour in the day might further increase anxiety and pain. However, the latter has no long-term effect on anxiety.

## Introduction

Septoplasty is one of the most frequently performed surgical procedures in an otolaryngology practice.^1^ The postoperative recovery time is relatively shorter for this minor surgical procedure, and the patient may be discharged before the postoperative 24th hour. Anesthesia techniques commonly used vary among sedation, local anesthesia and general anesthesia.^2^

Anxiety is an emotion characterized by feelings of tension and worried thoughts with an inexplicable cause. Anxiety and stress are among unpleasant feelings that may negatively affect the surgical operation and also the patient's recovery.^3^ According to previous publications, most of the patients admitted to the hospital for elective surgery experience preoperative anxiety.^4^, ^5^ The incidence of preoperative anxiety in adult patients was reported between 11% and 80%.^6^ The possible causes of preoperative anxiety might be getting away from home and relatives; disruption of daily routines; fear of loss of an organ or tissue, remaining disabled, inability of wake up at the end of surgery, pain after surgery and dying.^7^

Acute postoperative pain is defined as acute pain caused by the patient's pre-existing disease and/or surgical intervention he/she has undergone.^8^ Many patients experience severe pain after surgery or severe pain develops in the postoperative first hour, after arrival to the postoperative anesthesia care unit. Thomas et al. reported that analgesic requirements of the patients with high levels of anxiety increased in the postoperative period and pain control became difficult.^9^

Anxiety level might be affected by various factors like age, gender, failed surgical intervention, the size of the surgical procedure (minor/major), and operation time.^8^, ^10^ In this prospective-clinical study, we aimed to investigate the effects of the septoplasty operation and intraday operation time on anxiety and postoperative pain of the patients undergoing septoplasty.

## Methods

### Participants and study design

This prospective, clinical study was conducted in line with the dictates of the World Medical Association Declaration of Helsinki and approved by the local ethical committee of Aksaray University (IRB Number: E-18-2419). Among the patients admitted to the Ear- Nose-Throat outpatient clinic, 98 voluntary adult patients mentally capable of filling out a scale/questionnaire were included in our study. The patients who had a known psychiatric disease, a history of antidepressant medication use and a previous history of facial surgery and facial trauma were excluded from the study. To determine the anxiety levels of the patients, we applied the Hospital Depression and Anxiety Scale (HADS) to the patients for preoperative evaluation, three weeks before, one hour before the operation, and one week after the operation. The HADS is an assessment scale that evaluates the patient for anxiety and depression. It includes a total of 14 questions. Odd numbers measure anxiety, and even numbers measure depression. The Hospital Anxiety and Depression Scale (HADS) was developed by Zigmond and Snaith in 1983.^11^ The validity and reliability study of the Turkish version was performed by Aydemir et al.^12^

The difference between the HADS scores of the operation day (one hour before the operation) and the scores three weeks before the operation (difHADS) were calculated to determine the change in the HADS scores caused by the operation in the early period.

The patients were instructed to use Visual Analog Scale (VAS, Visual Analog Scale; 0: no pain; 10: most severe pain) one hour before the operation and in the postoperative first, sixth, twelfth and twenty-fourth hours to measure the pain levels, and the VAS scores of all patients were recorded. The VAS is an individual pain assessment method, and it is used to measure pain directly by the patient.^13^

No patient received a sedative or analgesic before the operation. All patients underwent surgery under general anesthesia with endotracheal intubation. Forty- nine patients underwent septoplasty at 8.00 am (morning group) and 49 underwent surgery at 03.00 pm (afternoon group). The preoperative fasting time of all patients was equal (7–8 h). The average operation time was 20–30 min. No complication was seen during or after the operations. In postoperative pain treatment, all patients received paracetamol (10 mg/kg) 4 times a day as the standard procedure, and we did not use an additional analgesic. All patients stayed in the hospital for twenty-four hours after the operation for short-term follow-up. The overall median HADS scores of the operation day were compared to the overall median HADS scores three weeks before and one week after the operation. To investigate the effects of the operation time on the anxiety level of the patients, the HADS scores three weeks before, one hour before and one week after surgery were compared between the morning group and the afternoon group. Moreover, difHADS values were compared between the morning group and afternoon group. In addition, we investigated the correlation between postoperative sixth-hour pain VAS scores and HADS scores one hour before surgery.

### Statistical analysis

Results are presented as median (min‒max). The abnormal distribution of data was confirmed using the Kolmogorov-Smirnov normality test (*p* <  0.05). To compare the overall HADS scores of the operation day (one hour before the operation) and the overall scores three weeks before the operation, Wilcoxon signed-rank test was used. To compare the HADS scores three weeks before, one hour before and one week after surgery, and to compare the difHADS between the morning group and the afternoon group, Mann-Whitney U test was used. To compare the pain VAS scores of the groups, Mann-Whitney U test was used as well. To investigate the correlation between the postoperative sixth-hour pain VAS scores and HADS scores one hour before surgery, the Spearman correlation test was used. All statistical analysis was performed using SPSS 16 software for Windows (SPSS Inc., Chicago, IL). A *p*-value under 0.05 was considered statistically significant.

## Results

Ninety-eight patients who underwent septoplasty were eligible for this study. Of these patients, 49 (24 males and 25 females, mean age: 30 ± 8 years) underwent septoplasty at 8.00 a.m. (the morning group), and 49 (25 males and 24 females, mean age: 31 ± 9 years) underwent septoplasty at 03.00 pm (the afternoon group). The groups were age and gender- matched (p = 0.67 and p = 0.84, respectively) (Table 1).

**Table 1:** Demographic variables of the study groups.

Variables	Morning group (n = 49)	Afternoon group (n = 49)	*p*-value
Age, years	30 ± 8	31 ± 9	*p* = 0.84
Gender (male/female)	24/25	25/24	*p* = 0.67

The comparison of the HADS scores of all patients at different times revealed that the median HADS scores one hour before the operation [6 (2–10)] were significantly higher compared to the median scores three weeks before the operation [3 (1–6)] (*p* <  0.001). On the other hand, the median HADS scores one week after the operation [2 (1–6)] were significantly lower compared to the median scores three weeks before the operation [3 (1–6)] (*p* <  0.001). Thus, we found that septoplasty operation had a significant short-term increasing effect on the anxiety levels of the patients regardless of the operation time, but the anxiety levels of the patients significantly decreased one week after surgery (Table 2).

**Table 2:** Comparison of HADS scores of all patients at different times.

	3 weeks before surgery	1 h before surgery	1 week after surgery	*p*-value
Median HADS scores	3 (1‒6)	6 (2‒10)	2 (1‒6)	*p* < 0.01^a^
				*p* < 0.01^b^

a Indicates the difference between the overall HADS scores 3 weeks before and 1 hour before the surgery.

b Indicates the difference between the overall HADS scores 3 weeks before and 1 week after the surgery.

The HADS scores of the morning group and afternoon group are presented in Table 3. The HADS scores three weeks before the operation (*p* =  0.767) and one week after the operation (*p* =  0.215) did not significantly differ between the groups. However, the HADS scores one hour before the operation were significantly greater in the afternoon group [8 (7–10)] compared to the morning group [4 (2–6)] (*p* <  0.001). Additionally, we found a significantly higher median difHADS in the afternoon group [5 (3–9)], compared to the morning group [0 (0–5)] (*p* <  0.001). Thus, we found that the late operation time had significantly more increasing effect on the anxiety levels of septoplasty patients (Table 3).

**Table 3:** Comparison of median HADS scores of the groups.

	Morning group	Afternoon group	*p*-value
HADS (3 weeks before surgery)	3 (1–4)	3 (1–6)	0.767
HADS (1 hour before surgery)	4 (2–6)	8 (7–10)	*p* < 0.01
HADS (1 week after surgery)	2 (1–4)	2 (1–6)	0.215
Difference between median HADS scores (3 weeks and 1 h before surgery)	0 (0–5)	5 (3–9)	*p* < 0.01

**Figure 1:**
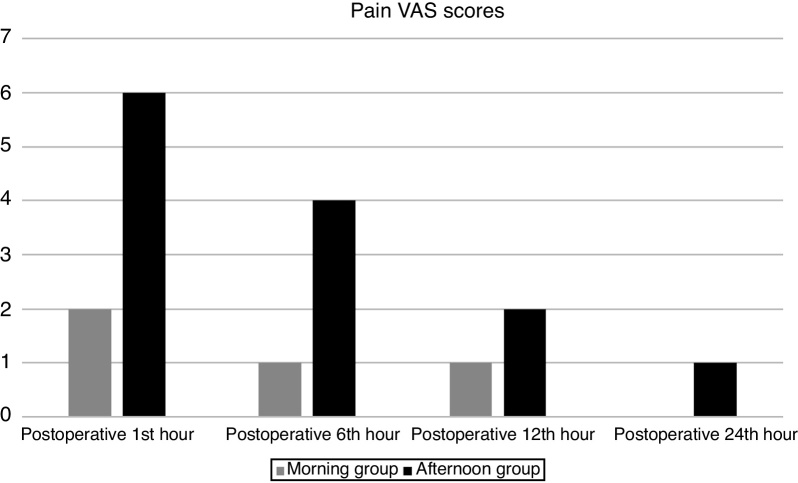
Median pain VAS scores of the groups.

The pain VAS scores of the groups were shown in Table 4. The pain VAS scores of all patients one hour before surgery were 0. Postoperative first, sixth, twelfth and twenty- fourth-hour pain VAS scores were significantly higher in the afternoon group compared to the morning group (*p* < 0.001) (Fig. 1). Thus, we found that the late operation time significantly increased the pain scores of septoplasty patients on the first day of the operation.

**Table 4:** Median pain VAS scores of the groups.

Pain VAS Score	Morning group	Afternoon group	*p*-value
One hour before surgery	0	0	‒
Postoperative 1st hour	2 (1‒4)	6 (3‒8)	<0.001
Postoperative 6th hour	1 (0‒3)	4 (2‒7)	<0.001
Postoperative 12th hour	1 (0‒1)	2 (1‒5)	<0.001
Postoperative 24th hour	0 (0‒1)	1 (1‒4)	<0.001

In addition, postoperative sixth-hour pain VAS scores were significantly (*p* < 0.001), positively, and strongly (rho = 0.78) correlated with the HADS scores one hour before the operation. Thus, we found that preoperative anxiety had a significant increasing effect on the pain levels of the patients undergoing septoplasty.

## Discussion

Preoperative anxiety negatively affects the surgery, anesthesia, and postoperative recovery process, by activating the release of neuroendocrine mediators and increasing the stress response. In the prior literature, anxiety was reported to be associated with some medical complications after surgery.^4^ According to Maranets and Kain, patients with anxiety need a higher dose of the anesthetic agent during surgery.^6^ The reduction of stress and anxiety might also reduce the risk of damage of the organs and complications, by decreasing the neurohormonal response to surgery.^7^, ^8^

Surgery has a known significant effect on anxiety levels of the patients. According to the previous publications, most of the patients underwent elective surgery had increased anxiety levels.^2^, ^4^, ^8^, ^10^ This increased anxiety might be associated with increased postoperative pain, higher postoperative analgesic requirement, and longer hospitalization.^3^, ^13^ A severe pain also has neurohormonal effects, such as increased sympathetic activity and increased stress hormones, and this may result in increased risk of postoperative complications like myocardial infarction or stroke due to the rupture of atherosclerotic plaques.^14^

Anxiety creates a physiological stress response undermining the healing process. It is known that surgery-related concerns may lead to anxiety even in minor surgical interventions not requiring long-term hospitalization.^8^ According to the report by Scott et al., 45.3% of patients underwent inpatient surgical intervention and 38.3% of outpatients had significant preoperative anxiety.^15^ In our study, the effects of septoplasty operation and operation time on anxiety and postoperative pain were evaluated. To the best of our knowledge, thus far no study in the English-language literature has investigated the effects of operation time on anxiety and postoperative pain.

Preoperative anxiety was reported to affect patient satisfaction and extend the duration of hospitalization.^4^, ^6^, ^8^ In our study, we compared the overall HADS scores of different times (three weeks before, one hour before and one week after surgery) to investigate the effect of septoplasty operation on anxiety levels. We found that the median HADS scores one hour before the operation were significantly higher compared to the median HADS scores three weeks before the operation. Furthermore, the median HADS scores one week after the operation were significantly lower compared to the median scores three weeks before the operation. Our results showed that septoplasty operation had a short-term increasing effect on anxiety levels, but the anxiety levels of patients significantly decreased one week after surgery. However, the mechanism of the decrease in anxiety levels one week after surgery is not clear. Although our data was not capable of demonstrating this mechanism, we can hypothesize that the relaxation due to the finished surgery and treated nasal obstruction might lead to the decrease in the anxiety levels one week after surgery.

According to our comprehensive English- language literature review, no study focusing on the association between operation time and anxiety was available. Badner et al., reported that preoperative afternoon anxiety was associated with the anxiety just before the operation.^4^ For the elective surgery planned, some patients might not accomplish the self- mental preparation process, resulting in increased anxiety. Furthermore, the length of the waiting period until the day of surgery may affect the level of anxiety.^16^ In our study, the HADS scores one hour before the operation were significantly greater in the afternoon group [8 (7–10)], compared to the morning group [4 (2–6)]. Additionally, we found that the median difHADS of the afternoon group [5 (3–9)] was significantly higher compared to the morning group [0 (0–5)]. Thus, we determined that the later operation time had a significantly greater effect on the anxiety levels of the patients undergoing septoplasty.

Many authors investigating preoperative anxiety levels concluded that patients with high levels of anxiety had more postoperative pain and used more analgesics.^13^, ^14^, ^17^ It was claimed that patients with higher anxiety scores felt more postoperative pain and needed more analgesics.^17^ According to the report by Ploghaus et al., anxiety increased the severity of pain, causing perceived discomfort greater than normal; and hippocampal formation was the factor that reduced the pain threshold by facilitating the activation of the entorhinal cortex.^18^ In our study, postoperative sixth-hour pain VAS scores were significantly (*p* <  0.001), positively, and strongly (rho = 0.78) correlated with the HADS scores one hour before surgery. Thus, we determined that preoperative anxiety had a significant increasing effect on the pain levels of the patients undergoing septoplasty.

Janis claimed that a lower preoperative anxiety level was associated with better postoperative recovery while a higher preoperative anxiety level was associated with poorer postoperative recovery.^19^ Kain et al. reported that the effect of the reduction of preoperative stress through sedation on the postoperative analgesic requirement and clinical recovery was minimal.^20^ In our study, we found that the pain VAS scores of all patients one hour before the operation were 0. We found that postoperative first; sixth, twelfth, and twenty-fourth hour pain VAS scores were significantly higher in the afternoon group compared to the morning group. This result showed that the late operation time significantly increased the pain scores of septoplasty patients on the first day of the operation. Increased postoperative pain might be due to increased anxiety levels in afternoon group; however, the main mechanism was not clear.

The main limitation of our study is the relatively small study population leading to the lack of generalization. In addition, administration of the scales more than once in a week- interval would provide more accurate results. However, our results suggest that minor surgical interventions should not be shifted to too later hours, to avoid complications regarding increased anxiety and pain. Furthermore, in addition to analgesics, anxiety reduction strategies should be implemented to decrease postoperative pain in the patients undergoing septoplasty.

## Conclusion

The results of this study suggest that the septoplasty operation, which is described as a minor surgery, might have an increasing effect on short-term anxiety and postoperative pain, and performing this operation at a late hour might further increase anxiety and pain. However, it has no effect on anxiety in the later period. Anxiety reduction strategies should be implemented to decrease postoperative pain in the patients undergoing septoplasty.

## Conflicts of interest

The authors declare no conflicts of interest.
